# Effect of Long-Duration Adventure Races on Cardiac Damage Biomarker Release and Muscular Function in Young Athletes

**DOI:** 10.3389/fphys.2020.00010

**Published:** 2020-02-05

**Authors:** Anthony Birat, Pierre Bourdier, Alexandre Dodu, Claire Grossoeuvre, Anthony J. Blazevich, Virgile Amiot, Anne-Charlotte Dupont, Stéphane Nottin, Sébastien Ratel

**Affiliations:** ^1^UFR STAPS – Laboratoire AME2P, Université Clermont Auvergne, Clermont-Ferrand, France; ^2^Fédération Française Triathlon, Saint-Denis, France; ^3^Centre for Exercise and Sports Science Research, School of Medical and Health Sciences, Edith Cowan University, Joondalup, WA, Australia; ^4^Service de Médecine du Sport et d’Exploration Fonctionnelle Respiratoire, Centre Hospitalier Régional, Orléans-La-Source, France; ^5^Imagerie Adaptative Diagnostique et Interventionnelle, CHRU de Nancy Brabois, Bâtiment Recherche, U1254 INSERM, Université de Lorraine, Nancy, France; ^6^Laboratoire de Pharm-Ecologie Cardiovasculaire, Avignon Université, Avignon, France

**Keywords:** muscle damage, cardiac impairment, wilderness multisport endurance events, adolescence, long duration exercise

## Abstract

The aim of the present study was to examine the effect of 1- and 2-day adventure races on cardiac muscle damage and skeletal muscle soreness and function in young athletes. Twelve male trained adolescents (14–15 years) completed both 1-day (48.2 km) and 2-day (66.0 km) races that included trail running, mountain biking, kayaking, and in-line skating separated by 10 weeks. Myocardial damage biomarker concentrations (cTnI and CK-MB), maximal voluntary isometric contraction (MVIC) torque, perceived knee extensor (KE) muscle soreness (PMS), and drop and squat jump heights were measured before and after each race. Heart rate was also monitored throughout. Mean heart rate (% cardiac reserve) was higher during the 1-day (66.6 ± 6.4%) than 2-day (62.6 ± 7.8%, *p* = 0.038) race. The amplitude of cardiac damage biomarker release was also higher following the 1-day than the 2-day race (peak cTnI: 0.14 vs. 0.03 ng/mL, *p* = 0.045; peak CK-MB: 20.30 vs. 11.98 ng/mL, *p* = 0.020). However, cardiac biomarker concentrations returned to baseline at 24–48 h post-exercise, except for CK-MB after the 2-day race (*p* = 0.017). Eight and three participants exceeded the cTnI cut-off for myocardial injury in 1- and 2-day races, respectively, but none exceeded the cut-off for acute myocardial infarction. While there was a significant decrease in drop jump height (−5.9%, *p* = 0.003), MVIC torque and squat jump height remained unchanged after both races. PMS was increased at 24 h after both races (*p* < 0.001) but returned to baseline levels by 72 h post-race. In conclusion, the shorter, more intense race produced more cardiac damage, although this probably represents a standard exercise intensity-dependent response rather than pathological response. Skeletal muscle functional and soreness responses were moderate and similar between races.

## Introduction

Over the last two decades, participation in long-duration adventure races (also called “wilderness multisport endurance events”) has become increasingly popular both in adolescents and adults. In these races, participants perform multiple activities including mainly trail running, mountain biking, kayaking, and orienteering. These events are held in wilderness environments and range in duration from a few hours (>4 h) to several days or weeks (Townes et al., 2004; McLaughlin et al., 2006) and might thus be expected to place significant stress on both cardiac and skeletal musculature. Whilst the impact of adventure races on the prevalence of injury and illness (Borland and Rogers, 1997) as well as inflammatory responses and oxidative stress (using blood biomarker analyses; de Lucas et al., 2014) have been examined in adults, limited data exist detailing the simultaneous effects of adventure races on the acute responses of cardiac and skeletal muscles in adolescents and adults. It is therefore unclear whether substantial strains on these tissues might be observed between these two populations.

Nonetheless, while clear scientific evidence is not yet available, these consequences might be expected to be greater during long-duration adventure races in adolescents compared to adults. This assumption is supported by the work of Tian et al. (2012) showing higher post-exercise peak troponin T (cTnT) concentrations after a 90-min treadmill run at 95% of the ventilatory threshold in 14-year-old adolescents than 24-year-old adults. This finding could be ascribed to the fact that at maximal exercise intensities, heart rate and total peripheral vascular resistance are higher in adolescents than adults while stroke volume and cardiac output are lower (Turley, 1997). These responses might disadvantage adolescent athletes with respect to myocardial work efficiency, which could trigger greater cardiac muscle damage biomarker release (e.g., troponins and creatine kinase-MB) than in adults, notably following long-duration endurance races (>4 h). Beyond the effects of age and maturation, these consequences could be exacerbated during shorter but more intense prolonged adventure races (<1 day) than during longer but less intense races (>1 day). This is supported by the recent systematic review and meta-analysis of Donaldson et al. (2019) showing a significant positive relationship between exercise intensity (i.e., average heart rate) and the rate of cardiac damage release (troponin) after exercise. However, the effect of race format (event duration vs. intensity) on the magnitude and temporal kinetics of cardiac damage biomarker release after long-duration adventure races (>4 h) in young adolescents remains to be demonstrated, particularly during field-based (rather than simulated, laboratory-based) competitions.

From a muscular perspective, mixed-modality, long-duration adventure races could also induce large reductions in skeletal muscle function of lower limbs in young adolescents. Indeed, it is well-known that the muscular force-generating capacity increases significantly during puberty (Tonson et al., 2010). This increase in force output is usually associated with structural changes, notably with skeletal muscle hypertrophy, higher type II fiber percentage and increased musculo-tendinous stiffness during adolescence (Lexell et al., 1992; Waugh et al., 2012). This could translate into increased skeletal muscle damage in adolescent athletes, particularly around growth spurt (Chen et al., 2014). This hypothesis is consistent with studies showing an increase of fatigability and skeletal muscle damage following eccentric-type activities (e.g., downhill running) from childhood to adulthood (Webber et al., 1989; Chen et al., 2014). Furthermore, the level of fatigability (i.e., force and power reductions and muscle soreness) of the lower-limb muscles could be greater during shorter but more intense prolonged adventure races in adolescents, as reductions in maximal force were found to be inversely associated with the duration of long-duration endurance races in adult athletes (Martin et al., 2010; Millet et al., 2011; Saugy et al., 2013). However, direct experimental evidence showing skeletal muscular consequences in response to different formats of long-duration adventure races during field-based competitions is not available in young adolescent athletes.

Therefore, the purpose of the present study was to evaluate the acute effects of mixed-modality endurance adventure races of different durations (1-day race vs. 2-day race) on cardiac and skeletal muscular responses in young adolescent athletes. In particular, we aimed to determine the amplitude and temporal kinetics of cardiac damage biomarker release (i.e., troponin I and CK-MB) after long-duration adventure races in the adolescent population. We hypothesized that the magnitude of cardiac biomarker release after races would decrease as the event duration increased due to the associated reduction in average exercise intensity and the resultant lesser impact on the cardiac system. We also postulated that shorter but more intense endurance adventure races (i.e., the 1-day race) would induce greater fatigability (i.e., force and power reductions) and muscle damage in lower-limb muscles than longer but less intense races (i.e., the 2-day race). However, the skeletal muscle damage was expected to be relatively minor compared to the magnitude of cardiac damage because of the mixed nature of the races, which included kayaking, cycling and in-line skating exercise that might induce less damage than running alone.

## Materials and Methods

### Subjects

Twelve male adolescents aged 14 to 15 years volunteered to participate in the present study. All participants trained in a local adventure race club 3 to 5 times per week in the 2 years preceding their participation in the experiments. During their weekly training, they mainly practiced endurance sports (trail running, mountain biking, kayaking, etc.) for 353 ± 84 min/week. They also regularly participated in national-level competitions. None of the participants had a family history of cardiovascular disease or was using any medication. This study was approved by an Institutional Ethics Review Board [Comité d’Éthique pour la Recherche en Sciences et Techniques des Activités Physiques et Sportives (CERSTAPS), no. 2018-08-06-24] and was conducted in conformity with the policy statement regarding the use of human subjects as outlined in the sixth *Declaration of Helsinki*. The young athletes were informed of the experimental procedures and gave their written assent before any testing was conducted. In addition, the written informed consent was obtained from the parents or legal guardians of the participants.

### Experimental Procedure (Design)

**FIGURE 1:**
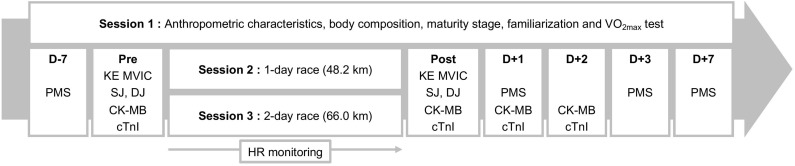
Experimental design. VO_2__max_, maximal O_2_ uptake; KE MVIC, maximal voluntary isometric contraction of the knee extensors; SJ, squat jump; DJ, drop jump; PMS, perceived muscle soreness; HR, heart rate. D-7, D + 1, D + 2, D + 3, and D + 7: measurements done 7 days before the race and on days 1, 2, 3, and 7 post-race, respectively.

The experimental design is illustrated in Figure 1. Briefly, volunteers were tested in three experimental sessions. The first session was dedicated to gathering participants’ physical characteristics (anthropometric measurements, body composition, and maturity status), maximal oxygen uptake (VO_2__max_) assessment and familiarization with the experimental procedures to be used during the second and third sessions. Furthermore, volunteers performed a series of maximal voluntary isometric contractions (MVIC) of the knee extensors (KE). They had to maximally contract their muscles at different knee joint angles (66, 80, and 92°; 0° = full extension) in a randomized order to determine the optimal angle for maximal KE torque production. During the second session, the volunteers participated in a competitive 42.7 km adventure race comprising multiple endurance-based activities (7.1 km of trail running, 27.0 km of mountain biking, 4.4 km of kayaking, and 4.2 km of in-line skating). This race was preceded the night before by a prologue of 5.5 km of trail running. The total distance covered by each participant was 48.2 km (“1-day race”). During the third session, the volunteers participated in a competitive race of 59.0 km on two successive days (Day 1: 32.0 km and Day 2: 27.0 km) by performing a series of mixed-modality endurance activities (14.0 km of trail running, 27.0 km of mountain biking and 18.0 km of kayaking). This race was preceded the night before by a prologue of 7.0 km of trail running. The total distance covered by each participant was 66.0 km (“2-day race”). The time interval between adventure races was 10 weeks.

To quantify the cardiac and skeletal muscular response to both races, measurements of cardiac muscle damage (capillary arterialized blood concentrations of cardiac troponin I [cTnI] and creatine kinase isoenzyme MB [CK-MB]) and skeletal muscle function (KE MVIC torque and both squat jump and drop jump heights) and perceived KE muscle soreness (PMS) were completed before and after each race (see below for further details). These experimental measurements were performed identically before and after both races. Furthermore, heart rate (HR) was continuously monitored during races using a Polar unit (V800, Polar, Kempele, Finland). Mean HR was calculated including both exercise and inter-exercise transition periods and then expressed as a percentage of heart rate reserve (HR_reserve_).

### Experimental Measurements

#### Session 1

##### Maximal O_2_ consumption (VO_2__max_) test

Each participant performed a progressive cycle test to exhaustion on a cycle ergometer (Cyclus model II, MSE Electronic Medical, Leipzig, Germany) to determine VO_2__max_. After a warm-up of 4 min at 80 W at 60 rpm, workload was increased by 20 W every minute until exhaustion. Oxygen uptake, carbon dioxide output, ventilation and heart rate were recorded with a CPX Jaeger OXYCON Pro (ACERTYS, Aartselaar, Belgium). Holter electrocardiogram was continuously monitored during the test. The end-point criteria used to validate the measurement of VO_2__max_ were (i) VO_2_ leveling off, (ii) maximal respiratory exchange ratio (RER_max_) ≥ 1.1, (iii) and maximal HR (HR_max_) ≥ 95% of the age-predicted HR_max_ (208.609–0.716 × age) (Shargal et al., 2015). Resting heart rate (HR_rest_) was measured early in the morning during a 10-min period of supine rest in a quiet place. Heart rate reserve (HR_reserve_) was calculated from the difference between HR_max_ and HR_rest_.

##### Anthropometric characteristics and body composition

Body mass was measured using a digital weight scale (TANITA, BC-545N, Japan) and standing height was assessed using a portable stadiometer with the participants barefoot (TANITA, HR001, Japan). Sitting height was also measured with the stadiometer while the participants sat on the floor with their back against a wall. Body mass index (BMI) was subsequently calculated using a standard formula, as follows: mass divided by height squared (kg.m^–2^). Skinfold thickness was measured in duplicate at the triceps and subscapular sites using a Harpenden caliper (Baty International, Burgess Hill, United Kingdom). The measurements were taken by the same investigator on the right side of the body to reduce variability in the results. Body fat (BF,%) was assessed using Slaughter’s equations (Slaughter et al., 1988).

##### Maturity status assessment

Maturity offset was used to assess somatic maturity and determined by using chronological age, height, sitting height and body mass. Its calculation was based on sex-specific regression equations according to the method proposed by Mirwald et al. (2002). Age at the peak height velocity was then calculated from chronological age and maturity offset.

#### Sessions 2 and 3

##### Maximal voluntary isometric contraction torque

Maximal voluntary isometric contraction torque of the dominant (preferred kicking) KE muscles was measured before and immediately after the end of each race using a custom-built ergometer, as described by Abdelmoula et al. (2012). This ergometer was built to adjust the force transducer at the level of the lateral malleolus and change the seat depth depending on thigh length. The hip angle was set at 40° (0° = full extension). The knee was fixed at the optimal angle (76.3 ± 8.3°) for maximal force production, which was determined during the first session (see above). Volunteers were secured to the chair by a strap over their shoulders to minimize compensatory trunk movement. They were also instructed to grip the seat during the voluntary contractions to further stabilize the pelvis. During each trial, volunteers were instructed to contract as strongly as possible. The same settings were used for each individual before and after each race. Force data were measured using a calibrated force transducer (Model F2712, 0- to 1000-N force range, Meiri Company, Bonneuil-sur-Marne, France) connected to an ankle cuff and exported at a rate of 2 kHz to an external analog-to-digital converter (PowerLab 8/35, ADInstrument, Bella Vista, NSW, Australia) driven by LabChart 7.3 Pro software (ADInstrument). Visual feedback of force as well as verbal encouragement during MVIC were provided to the volunteer by the same experimenter. Isometric KE MVC torque was calculated as the product of maximal force and moment arm length, the latter being measured from the lateral malleolus to the lateral femoral condyle in resting conditions using a tape meter (Abdelmoula et al., 2012).

##### Jumping performance

Squat jump and drop jump tests were performed before and immediately after completion of each race. They were performed using an optical measurement system (OptoJump, Microgate, Bolzano, Italy) with the following instructions.

###### Squat jump (SJ)

From a standing position, the participants flexed their knees to 90° to lower their center of mass (i.e., to a squatting position). This position was held for 3 s before a maximal vertical jump was performed without countermovement. The jump was repeated if the experimenter could clearly, visually detect a countermovement prior to the upward (jump) phase. This test was used as an indicator of dynamic muscle power without use of a stretch-shorten cycle.

###### Drop jump (DJ)

A box of 40 cm height was placed in front of the OptoJump system. From a standing position close to the front edge of the box, the subjects extended one foot out in front of themselves to allow their body to move forward over the edge of the box before letting their body fall vertically without pushing upwards or outwards, jumping or flexing their support leg from the box; when done properly, the subjects landed close to the box (heel < 25 cm from the front of the box). Upon landing with both feet, they immediately jumped as high as possible. In their native French language, the instruction to the children was to “push as strong as possible and reduce the ground contact time.” This test was used as an indicator of dynamic muscle power when an intense eccentric loading of the body is applied prior to concentric muscle contraction.

In all jump tests, the hands were placed on the hips with the elbows bowed outward (akimbo position). For all jumps, jump height was derived from the flight time of the participants.

The two jump types (one including and the other not including stretch-shorten cycle activity) as well as KE MVIC were chosen to evaluate the effects of both adventure races on muscle function (Byrne and Eston, 2002).

##### Perceived muscle soreness (PMS)

Perceived muscle soreness (PMS) score of the KE muscles was evaluated 7 days before (D-7) and on days 1 (D + 1), 3 (D + 3), and 7 (D + 7) after completion of each race by using a visual analog scale consisting in a horizontal line graduated from 0 (“no pain”) to 10 (“very, very sore”). Participants were asked to place a vertical mark on the horizontal line to describe the pain experienced during a slow, unloaded knee flexion movement to a 90° angle. The distance between the origin of the scale and the vertical mark was used as the pain score.

##### Biomarkers of cardiac damage

Capillary arterialized blood samples (100 μl) were drawn from the earlobe and collected in heparinized tubes before (Pre), immediately after (Post) and on days 1 (D + 1) and 2 (D + 2) following each race to determine the time course of cTnI and CK-MB. The myocardial damage biomarkers concentrations were analyzed using the i-STAT^®^ handheld analyzer (Abbott Point of Care, Inc., Princeton, NJ, United States), which was clinically validated (Pickering et al., 2018). The measurements were immediately made after collection using i-STAT cTnI and CK-MB cartridges. The reportable range is 0.0–50.0 ng/mL for cTnI and 0.0–150.0 ng/mL for CK-MB. Diagnosis of the potential risk of exercise-induced myocardial injury was done by comparing the post-exercise maximal value of cTnI with the myocardial injury cut-off of 0.06 ng/mL, according to the guidelines of *European Society of Cardiology and American College of Cardiology* (Alpert et al., 2000) and the acute myocardial infarction (AMI) cut-off of 0.50 ng/mL (Collinson and Chamberlain, 2001).

### Statistical Analysis

Data were screened for normality of distribution and homogeneity of variances using a Shapiro–Wilk normality test and the Barlett’s test, respectively. The exercise duration and mean heart rate (HR_*mean*_) were compared between adventure races using a paired Student’s *t*-test. Non-parametric Friedman’s test was used to analyze the changes in the cardiac biomarkers across the time points (Pre, Post, D + 1, D + 2) because of the skewed distribution of the CK-MB and cTnI concentrations. Wilcoxon signed-ranks test was completed for pairwise comparisons when Friedman’s test revealed a significant effect. CK-MB and cTnI concentrations were compared between races at each time point using Mann–Whitney *U*-test. Two-way (race format × time) ANOVA with repeated measures was used to examine the effect of race format (1-day race vs. 2-day race) on changes in KE MVIC torque, SJ and DJ heights and PMS. When ANOVA revealed a main or interaction significant effect, a Fisher’s LSD *post hoc* test was applied to test the discrimination between means. The effect size and statistical power have also been reported when significant main or interaction effects were detected. The effect size was assessed using the partial eta-squared (η^2^) and ranked as follows: ∼0.01 = small effect, ∼0.06 = moderate effect, ≥ 0.14 = large effect (Cohen, 1969). The limit for statistical significance was set at *p* < 0.05. Statistical procedures were performed using Statistica 8.0 software (Statsoft, Inc., United States). Results are presented in the text and tables as mean ± SD.

## Results

### Participants’ Physical and Fitness Characteristics

Participants’ characteristics are described in Table 1. All the boys were circa-pubertal. Before the races, no participant showed any abnormal electrocardiogram.

**TABLE 1:** Participants’ physical and fitness characteristics.

**Variable**	**Participants (*n* = 12)**
Age (years)	14.40.5
Maturity offset (years)	0.20.8
APHV (years)	14.20.7
Height (m)	1.660.08
Body mass (kg)	54.08.1
Body mass index (kg.m^–2^)	19.61.9
Body fat (%)	13.52.8
VO_2__max_ (mL.kg^–1^.min^–1^)	66.86.9
HR_max_ (bpm)	202.38.6
HR_rest_ (bpm)	64.87.9
HR_reserve_ (bpm)	137.38.9

*Values are presented as mean ± SD. APHV: age assessed at the peak height velocity, VO_2__*max*_: maximal O_2_ uptake, HR_*max*_: maximal heart rate, HR_*rest*_: resting heart rate, HR_*reserve*_: heart rate reserve.*

### Adventure Races Features

As expected, the mean completion time was significantly shorter during the 1-day race than the 2-day race (334 ± 42 vs. 560 ± 42 min, *p* < 0.001). Furthermore, HR_mean_ expressed as a percentage of HR_reserve_ was significantly higher during the 1-day race than the 2-day race (66.6 ± 6.4 vs. 62.6 ± 7.8%HR_reserve_, *p* = 0.038).

### Cardiac Damage Biomarker Release

**FIGURE 2:**
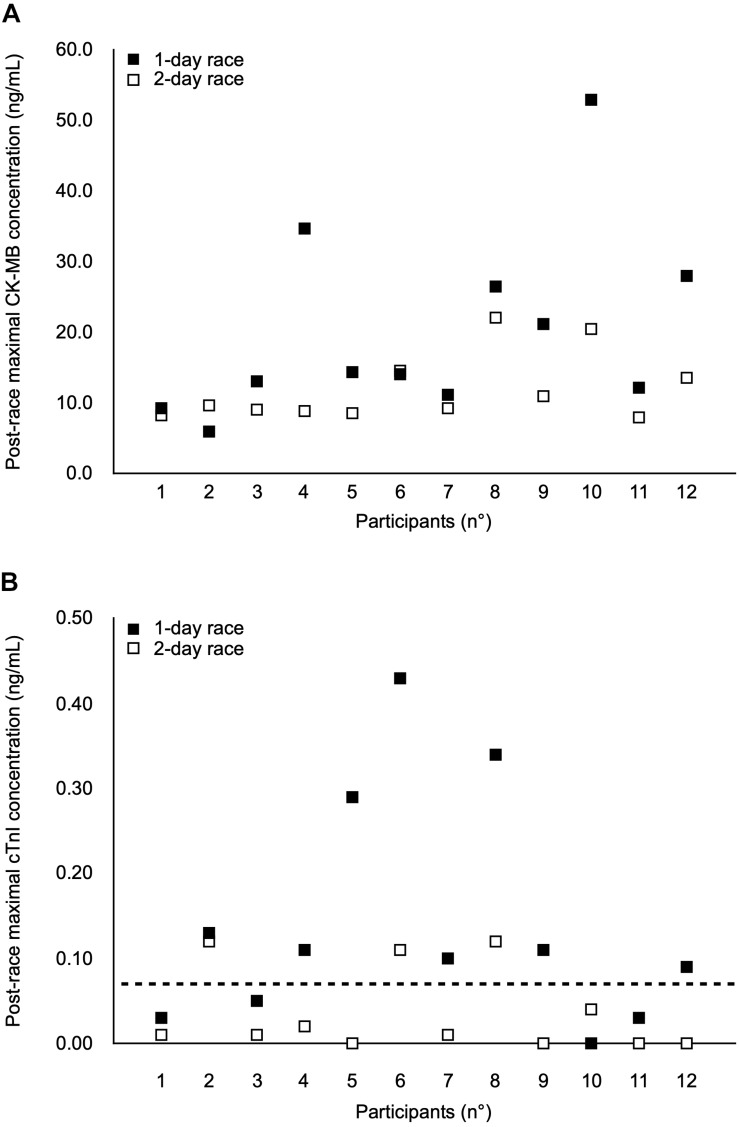
Individual post-race maximal values of creatine kinase-MB (CK-MB) **(A)** and cardiac Troponin I (cTnI) **(B)** in 12 adolescent male athletes after the 1-day race and the 2-day race. The horizontal dotted line represents the cTnI cut-off of 0.06 ng/mL for myocardial injury (Alpert et al., 2000). Each participant is represented vertically.

The time courses of changes in CK-MB and cTnI concentrations during and following both races are shown in Table 2, and post-race maximal individual CK-MB and cTnI values are indicated in Figures 2A,B, respectively. CK-MB and cTnI reached peak values immediately or 1 day after both races, and values were approximately 2- and 4-fold higher following the 1-day race than the 2-day race, respectively (CK-MB, *p* = 0.020, cTnI: *p* = 0.045). However, cardiac biomarker concentrations returned to baseline by D + 2 after completion of both races except for CK-MB after the 2-day race (*p* = 0.017) (Table 2).

**TABLE 2:** Creatine kinase-MB (CK-MB) and cardiac troponin I (cTnl) concentrations obtained before (Pre), immediately after (Post) and days 1 (D + l) and 2 (D + 2) following each race.

	**1-day race**				**2-day race**			
	**Pre**	**Post**	**D + 1**	**D + 2**	**Pre**	**Post**	**D + 1**	**D + 2**
**CK-MB (ng/mL)**								
Mean	4.04	20.30**	15.24**	5.77	3.51	11.98** ^$^	6.87** ^$$^	5.95*
SD	2.61	12.81	8.83	3.52	1.70	4.62	2.77	3.18
Minimum–maximum	1.40–11.10	6.00–52.90	4.70–35.30	2.60–14.80	1.60–7.40	8.00–22.10	3.80–13.00	3.10–14.00
**cTnI (ng/mL)**								
Mean	0.004	0.143**	0.033**	0.013	0.003	0.037* ^$^	0.007 ^$$^	0.006
SD	0.005	0.131	0.030	0.020	0.006	0.047	0.010	0.009
Minimum–maximum	0.000–0.010	0.000–0.430	0.010–0.100	0.000–0.070	0.000–0.020	0.000–0.120	0.000–0.030	0.000–0.030

Values are presented as mean ± SD. *, **: significantly different from Pre at *p* < 0.05, and *p* < 0.01, respectively, $, $$: significantly different from the 1-day race at the same time at *p* < 0.05 and *p* < 0.01, respectively.

No participant exceeded the cTnI cut-off value of 0.06 ng/mL for myocardial injury when assessed before the races. However, 8 out of 12 participants (66.7%) exceeded value immediately after the 1-day race, with two participants remaining above the value at D + 1. Immediately after the 2-day race, only 3 out of 12 participants (25.0%) exceeded the cut-off. Two of them systematically exceeded this cut-off after both races. No participant exceeded the cTnI cut-off of 0.50 ng/mL for AMI after either race.

### Muscle Function and Soreness

No participants reported any muscle injury or health problems during or following the races. ANOVA showed no significant race format × time interaction effect for KE MVIC torque, SJ and DJ heights or PMS (Table 3). ANOVA also revealed no significant time effect for KE MVIC and SJ height (Table 3). However, there was a significant time effect for DJ height [*F*_(__1_,_11__)_ = 13.8, *p* = 0.003, η^2^ = 0.56, power = 0.92], whereby both race formats induced a significant decrease in DJ height by an average of 5.9% (Table 3). There was also a significant time effect for PMS [*F*_(__3_,_36__)_ = 10.3, *p* < 0.001, η^2^ = 0.46, power = 0.99], which was significantly increased from D-7 (0.96 ± 1.59) to D + 1 (2.96 ± 1.84, *p* < 0.001) and then returned to baseline values by D + 3 (1.12 ± 1.03) and D + 7 (0.85 ± 1.26).

**TABLE 3:** Maximal voluntary isometric contraction torque of knee extensors (KE MVIC torque) and squat jump (SJ) and drop jump (DJ) heights obtained before (Pre) and immediately after (Post) each race.

	**1-day race**		**2-day race**		***F*-values (*p-*values)**		
	**Pre**	**Post**	**Pre**	**Post**	**Race format**	**Time**	**Race format × time interaction**
KE MVIC torque (N.m)	208.1 ± 49.2	194.9 ± 44.4	239.0 ± 51.1	227.8 ± 56.4	*F*(1;11) = 1.6 (0.230)	*F*(1;11) = 3.3 (0.099)	*F*(1;11) = 0.1 (0.831)
SJ height (cm)	29.5 ± 4.7	26.7 ± 4.5	30.3 ± 5.1	29.7 ± 2.7	*F*(1;11) = 2.5 (0.142)	*F*(1;11) = 4.3 (0.063)	*F*(1;11) = 1.0 (0.236)
DJ height (cm)	28.8 ± 4.2	26.6 ± 4.3	30.9 ± 3.1	29.4 ± 3.1	*F*(1;11) = 2.7 (0.127)	*F*(1;11) = 13.8 (0.003)	*F*(1;11) = 0.5 (0.236)

*Values are presented as mean ± SD.*

## Discussion

The aim of the present study was to evaluate the effects of mixed-modality, long-duration adventure races during field-based competitions on cardiac and skeletal muscle responses in adolescent male athletes and to determine whether these effects differed as a function of race duration (1-day race vs. 2-day race). The main results were consistent with our hypotheses since biomarker analysis indicated that the magnitude of cardiac damage in trained adolescent athletes was greater following the shorter endurance adventure race (1-day race) than the longer race (2-day race). Nonetheless, while cTnI concentrations exceeded the 0.06 ng/mL definition of myocardial injury immediately after 1- (66.7%) and 2-day (25.0%) races, no participant exceeded the 0.50 ng/mL definition for AMI. Additionally, both races had little effect on lower-limb (skeletal) muscular function, and induced only moderate levels of perceived knee extensor muscle soreness within 24–72 h of racing. Importantly, no participant reported physical injury or health problems during or following the races.

### Cardiac Damage Biomarker Release

While the cardiac consequences of exercise of different modalities have previously been studied in children and adolescents, notably following intermittent intense activities, e.g., football, basketball, and table tennis (Nie et al., 2008; Ma et al., 2014; Lopez-Laval et al., 2016; Hosseini et al., 2018) or 60-min continuous exercise (Legaz-Arrese et al., 2017), few studies have investigated serological release of cardiac damage biomarkers in response to long-duration endurance races (>4 h) in adolescent athletes. The results of the present study clearly show that long-duration adventure races stimulated cardiac muscle damage biomarker release, as illustrated by significantly increased post-exercise concentrations of CK-MB and cTnI (see Table 2). Nonetheless, the 1- and 2-day races elicited lower peak cTnI values than half-marathon and marathon races (Tian et al., 2006; Traiperm et al., 2012), and cTnI concentrations returned to baseline faster after both long-duration adventure races than after half-marathon and marathon races (Tian et al., 2006; Traiperm et al., 2012). These findings are consistent with the belief that elevated concentrations of cardiac damage biomarkers reflect acute physiological rather than pathological responses to exercise in young athletes. In fact, any exercise-induced release of cardiac damage biomarkers in young athletes without clinical suspicion of a cardiac problem might indicate transient cytosolic leakage propagated by membrane damage, rather than cardiomyocyte necrosis (Shave et al., 2004). Importantly, adventure racing appears to impose a relatively low risk to cardiac tissue in adolescent athletes.

In the present study, the magnitude of cardiac damage biomarker release was greater following the shorter but more intense adventure race (1-day race) compared to the longer and less-intense format (2-day race). Peak CK-MB and cTnI values were about 2- and 4-fold higher following the 1-day race than the 2-day race, respectively. This outcome is consistent with findings of Fu et al. (2009) showing that exercise intensity rather than duration appeared to cause a more pronounced cardiac damage biomarker release during 45- and 90-min constant-load treadmill runs at different intensities. However, a delimitation of the present data is that it was not possible to obtain blood after day 1 of the 2-day race. Whilst it is clear that cardiac damage at the end of the 2-day race was less than in the 1-day race, it cannot be completely discounted that damage was the same after day 1 and then subsided by day 2. Certainly, some protective (i.e., repeated bout) effects have been observed in cardiac damage biomarkers when a 4-h rest was given between two bouts of 45 min of running in trained adolescent runners (Nie et al., 2011). Such data indicate the likelihood of a “cardio-protective mechanism” of an initial exercise bout. Nonetheless, participants in the present study were experienced endurance athletes who completed trail running, mountain biking, kayaking, etc., for 353 ± 84 min/week, and this training should already have provided some protection from cardiac damage; an additional day of exercise might therefore be expected to have less effect in this population. Furthermore, the lower level of damage observed after the 2-day race compared to the 1-day race is consistent with the hypothesis that, at least when a sufficient duration of exercise is performed, lower intensities of exercise are associated with lower levels of cardiac damage (Donaldson et al., 2019). The results of the present study might therefore be considered a good reflection of the damage expected after each day of racing, although the purpose of the study was specifically to determine the level of damage after the whole competition.

### Skeletal Muscle Function and Damage

The present results also show that mixed-modality adventure races might induce little or no alteration in MVIC capacity of skeletal muscles (the knee extensors in the present study), although a significant and moderate increase in PMS was observed in the 24–48 h period following both races. The lack of change in isometric muscle strength associated with a significant increase in PMS after both races was unexpected since delayed onset muscle soreness is often associated with the loss of force after long-distance running-based races (Saugy et al., 2013). However, some authors have shown that PMS is more related to the increase in inflammatory markers in the epimysium (e.g., after 45 min of downhill running) than in the skeletal muscle fibers themselves, thereby suggesting greater damage in the connective tissue or fascia (Malm et al., 2004). Lau et al. (2015) also showed that PMS was more associated with damage inflammation to fascia than muscles fibers after eccentric exercise. The results of the present study concur with these findings (i.e., greater fascia damage); however, direct scientific evidence showing such relationship between muscular function and the origins of PMS still remains to be demonstrated in young athletes. Furthermore, squat jump height, taken to reflect maximal dynamic muscle power, was not affected by the racing, whilst drop jump performance was significantly but only moderately (5.9%) reduced. Differences in muscle activation patterns and levels of peripheral (afferent) feedback between the isometric and concentric versus stretch-shorten cycle tasks might underpin the differences in functional loss identified by the tests. Contrary to isometric and concentric contractions, performances in eccentric contractions may be particularly prone to exercise-induced muscle damage (Kellis and Baltzopoulos, 1998). This is illustrated in the present study through the absence of changes in maximal isometric KE torque and SJ height despite a statistical decrement in DJ height being observed.

For comparison, both of the adventure races studied presently induced relatively less alteration in muscular function than the most extreme trails or ultra-endurance running. In fact, some studies have reported significantly greater KE force losses after a mountain-based ultra-marathon of 330 km (−23%; Saugy et al., 2013) or a 24-h treadmill run (−41%; Martin et al., 2010) in adult athletes. Moreover, Saugy et al. (2013) reported that perceived muscle soreness exceeded 6 on a 10-point scale after a mountain-based ultra-marathon, while in the present study PMS did not exceed 3 out of 10 in either race. These differences might speculatively be explained in several ways. Firstly, there was a lesser requirement for eccentric muscle contractions in the present study (where there was minimal downhill running) than the study of Saugy et al. (2013) where there was a significant volume of downhill running. In fact, in the present study only 17 and 32% of the total distance consisted of trail running during the 1- and 2-day races, respectively, with the remainder of the races requiring the use of other exercise modes such as mountain biking and in-line skating, which predominately involve concentric muscle actions and thus induce less lower-limb skeletal muscle damage (Stocchero et al., 2014), or kayaking in which the lower body muscles may participate less. Secondly, exercise-induced muscle damage is age-dependent. In fact, Webber et al. (1989) found that children produced lower creatine kinase levels after a single bout of downhill running (30 min, −10% grade) than adults, possibly because of their lower body mass, lesser ability to generate force per unit fiber area, lower musculotendinous stiffness, and lower percentage of type II fibers, which tend to be more susceptible to damage (Lexell et al., 1992; Waugh et al., 2012). These factors might have contributed to the minimal decrease of muscle function in the adolescent athletes after both races; however, this assertion remains to be confirmed.

Several limitations should be mentioned in this study. We only studied 12 male adolescent athletes. However, this small sample size should not affect the conclusions of the present study since effect sizes and statistical power were high. Another point of consideration is that female adolescent athletes were not evaluated. Thus, it remains to be determined whether cardiac or skeletal muscular responses after long-duration adventure races are consistent in adolescents regardless of sex. Further studies should address such issues by recruiting female adolescent athletes. Finally, no evaluation of cardiac function was done in the current study. Additional measures of left ventricular function using Doppler echocardiography could be done in association with the evaluation of blood cardiac damage biomarkers to further investigate the effect of long-duration adventure races on cardiac fatigue and damage and their link during adolescence.

## Conclusion

The shorter but more intense 1-day adventure race produced more cardiac muscle damage than the longer but less intense 2-day race; however, biomarkers of cardiac damage (cTnI and CK-MB) returned to baseline within 24–48 h of both races except for CK-MB after the 2-day race and no participant exceeded the cTnI cut-off of 0.50 ng/mL for AMI. With regard to skeletal muscle damage and function, both races induced minimal alteration in lower-limb muscle function and produced only moderate levels of perceived knee extensor muscle soreness. These results suggest that prolonged exercise in adolescents might trigger acute physiological responses but not a pathological response, and that long-distance multi-sport adventure races probably pose minimal acute cardiac and skeletal muscle risk. However, while it was clarified that adolescents can perform long periods of exercise without appreciable loss of cardiac or skeletal muscle function, the long-term effects of repeated prolonged adventure races on other processes such as left ventricular function remain to be examined in young athletes.

## Data Availability Statement

The raw data supporting the conclusions of this article will be made available by the authors, without undue reservation, to any qualified researcher. Requests should be directed to sebastien.ratel@uca.fr.

## Ethics Statement

This study was approved by an Institutional Ethics Review Board [Comité d’Éthique pour la Recherche en Sciences et Techniques des Activités Physiques et Sportives (CERSTAPS), no. 2018-08-06-24] and was conducted in conformity with the policy statement regarding the use of human subjects as outlined in the sixth Declaration of Helsinki. The young athletes were informed of the experimental procedures and gave their written assent before any testing was conducted. In addition, the written informed consent was obtained from the parents or legal guardians of the participants.

## Author Contributions

This study was conducted in the laboratory of metabolic adaptations during exercise in physiological and pathological conditions (AME2P, EA 3533) at the Clermont Auvergne University, France. AB, AD, and SR designed the research. AB, PB, AD, CG, VA, and SR collected the data and performed the research. AB, AD, and SR analyzed the data and supervised the research. AB, AJB, and SR wrote the manuscript. AB, AJB, PB, AD, CG, VA, A-CD, SN, and SR provided critical revisions important for intellectual content of the finished manuscript, approved the final version of the manuscript, and agreed to be accountable for all aspects of the work in ensuring that questions related to the accuracy or integrity of any part of the work are appropriately investigated and resolved. All persons designated as authors qualify for authorship, and all those who qualify for authorship are listed.

## Conflict of Interest

The authors declare that the research was conducted in the absence of any commercial or financial relationships that could be construed as a potential conflict of interest.
